# Supplementary material for: Content-rich biological network constructed by mining PubMed abstracts
Source: BMC Bioinformatics. 2004 Oct 8;5:147. doi: 10.1186/1471-2105-5-147 (PMC528731; doi:10.1186/1471-2105-5-147)
Supplement: Additional File 2 — The original results of the above study (non-essential files are deleted to keep the file size under the limit set by BMC bioinformatics). [file 1471-2105-5-147-S2.bz2 › chilibotAdditionalFile2/dip10/405ID10681548E10065/html/IL6_OSM.html]

 


 **IL6** and **OSM** 
  
Found 446 abstracts in PubMed, retrieved 10.  
 

 What does Google say? 
 PDF only 
| .edu only 

---

**Interactive relationship** (e.g. stimulation, inhibition, etc)

**Neutral relationship**- Oncostatin M  [ **OSM** ]  belongs to the interleukin 6  [ **IL6** ]  family of cytokines and acts as a multifunctional cytokine during murine embryogenesis and in inflammatory reactions.  Ref: 12814362 Eur J Neurosci, 2003
- Oncostatin M  [ **OSM** ]   **OSM**  is a multifucntinal cytokine that belongs to the IL 6  [ **IL6** ]  family.  Ref: 12855584 Blood< MedlineTA>Blood, 2003
- Oncostatin M  [ **OSM** ]   **OSM**  is a multifunctional cytokine that belongs to the Interleukin IL 6  [ **IL6** ]  subfamily.  Ref: 12811586 Rev Physiol Biochem Pharmacol< MedlineTA>Rev Physiol Biochem Pharmacol, 2003
- Oncostatin M  [ **OSM** ]   **OSM**  is a member of the interleukin 6  [ **IL6** ]  cytokine family, which is involved in definitive hematopoiesis, the development of liver, and local inflammation.  Ref: 12831858 Neuroscience, 2003
- CONCLUSION IL 1, but not IL 6  [ **IL6** ] , TNFalpha, or iNOS, plays an important role in joint disease induced by intraarticular gene transfer of  **OSM**  in mice.  Ref: 12794844 Arthritis Rheum, 2003
- METHODS An adenoviral vector expressing murine  **OSM**  was injected into the joints of naive wild type mice and mice deficient for interleukin 1 IL 1, IL 6  [ **IL6** ] , tumor necrosis factor alpha TNFalpha, or inducible nitric oxide synthase iNOS.  Ref: 12794844 Arthritis Rheum, 2003
- IL 6  [ **IL6** ]  type cytokines exert their action via the signal transducers gp130, LIF receptor and  **OSM**  receptor leading to the activation of the JAK STAT and MAPK cascades.This review focuses on recent progress in the understanding of the molecular mechanisms of IL 6  [ **IL6** ]  type cytokine signal transduction.  Ref: 12773095 Biochem J< MedlineTA>Biochem J, 2003

**Non-interactive relationship** (e.g. studied together, co-existance, homology, etc.)

- The IL 6  [ **IL6** ]  family cytokine oncostatin M  [ **OSM** ]  was also capable of activating MAPK, STAT3, STAT1, Akt, and p38 in both WT and SYF cells.  Ref: 12764151 J Biol Chem< MedlineTA>J Biol Chem, 2003
- Studies with cultured cells and mouse models of disease have ascribed proinflammatory and anti inflammatory functions to oncostatin M  [ **OSM** ]   **OSM**  and the related cytokine, interleukin 6  [ **IL6** ]  IL 6  [ **IL6** ] .  Ref: 12856331 J Interferon Cytokine Res, 2003
- Here, we examined the effect of systemic administration of adenoviral Ad vectors encoding either murine  **OSM**  AdMuOSM or murine IL 6  [ **IL6** ]  AdMuIL 6 in a mouse model of colitis.  Ref: 12856331 J Interferon Cytokine Res, 2003
- after IL 6  [ **IL6** ]  and  **OSM**  stimulation, gp130 dependent pathways STAT3, APR gene expression were completely blocked in the liver of these animals.  Ref: 12891556 Gastroenterology, 2003
- Hepatocyte specific gene expression in BMCs was identified by immunocytochemistry and reverse transcription polymerase chain reaction.RESULTS Bone marrow cell derived hepatocyte like colonies appeared after several days of coculture in medium containing HSLF, oncostatin M  [ **OSM** ]   **OSM**  and hepatocyte growth factor HGF  [ **IL6** ] .  Ref: 12821039 J Hepatol, 2003
- Adult mice were challenged with IL 6  [ **IL6** ] , oncostatin M  [ **OSM** ]   **OSM** , and LPS.  Ref: 12891556 Gastroenterology, 2003
- Transdifferentiation was enhanced by 5 azacytidine treatment, and by HSLF,  **OSM**  and HGF  [ **IL6** ] .  Ref: 12821039 J Hepatol, 2003
- The IL 6  [ **IL6** ]  type cytokines IL 6  [ **IL6** ] , IL 11, LIF,  **OSM** , CNTF, CT 1 and CLC are an important family of mediators involved in the regulation of the acute phase response to injury and infection.  Ref: 12773095 Biochem J< MedlineTA>Biochem J, 2003
